# A Case of Amoebic Liver Abscess Presenting as a Therapeutic Conundrum Necessitating the Exploration of Other Under-the-Radar Treatment Modalities

**DOI:** 10.7759/cureus.28493

**Published:** 2022-08-28

**Authors:** Kushagra Khanna, Rajika Khanna, Sourya Acharya, Sunil Kumar

**Affiliations:** 1 Department of Medicine, Jawaharlal Nehru Medical College, Datta Meghe Institute of Medical Sciences (Deemed to be University), Wardha, IND; 2 Department of Medicine, Kasturba Medical College, Manipal Academy of Higher Education, Manipal, IND

**Keywords:** therapeutic dilemma, non-conventional treatment modalities, percutaneous drainage, dual attack drug therapy, entamoeba histolytica

## Abstract

Tropical countries have recorded incidences of amoebic liver abscess (ALA) owing to the more significant epidemiology and pathogenicity of the intestinal protozoan Entamoeba histolytica in these regions^.^ However, the rise in the number of immigrants from such areas to developed countries has made it necessary to thoroughly review the diseases caused by the parasite globally. Patients generally present with the usual features of right upper quadrant pain, painful hepatomegaly, mild jaundice, and vomiting, to name a few; however, the condition can manifest varied presentations accompanied by a plethora of findings non-responsive to the standard treatment. Therefore, newer modalities should be considered by weighing their risks and benefits to reduce morbidity and mortality and improve patient outcomes.

## Introduction

Less than 1% of those with Entamoeba histolytica infection develop an amoebic liver abscess (ALA), the most common extraintestinal location of amoebic infection [1]. Males are more commonly affected than females, and alcohol use [2] has been found to be a significant risk factor. Though the disease is treatable and has a good prognosis, it can lead to morbidity and even mortality if the treatment is delayed [3]. Anti-amoebic therapy alone can be effective in treating uncomplicated abscesses. However, this regimen is ineffective against complicated cases, which require additional drainage through a radiologically guided percutaneous pigtail catheter. Most infected patients have shown marked improvement in the signs and symptoms following such treatments, but a few fail to respond to them, necessitating more invasive methods such as surgical drainage [4-6]. The crux of the case we present in this study involves residual disease despite the vigorous initial treatment; it had required surgery, which was deferred, causing the patient to develop a chronic hepatic abscess later. This eventually led to the exploration of other treatment routes, which could have prevented long-term complications. To avoid residual amoebiasis from wreaking havoc after treatment, thorough post-discharge follow-up and evaluation of non-conventional options available could prove beneficial.

## Case presentation

A 45-year-old male patient, a chronic alcoholic, presented to the emergency room with dyspnea, repeated vomiting episodes starting one night prior, and jaundice lasting four days. The patient had dull aching pain in the right upper quadrant of the liver, breathlessness, and cough with white expectoration for two months. There was no history of any comorbidities. At the time of examination, the patient was drowsy and had a diminished verbal reaction with a Glasgow Coma Scale score of 10, pulse rate of 96/minute, blood pressure of 140/90 mmHg, respiratory rate of 24/minute, SpO_2 _of 100% on non-invasive ventilation (NIV) positive end-expiratory pressure (PEEP) of 5 cmH_2_O with the presence of conjunctival pallor and mild pedal edema. On percussion, a dull note was heard in the right infra-axillary and right eighth intercostal space, and right infrascapular areas. Auscultation of the right infra-axillary and infra-scapular areas revealed reduced breath sounds.

An abdominal examination revealed tenderness and distension with a 16-cm liver span. The patient was admitted to the ICU, and IV crystalloid fluid resuscitation and empiric antibiotics were started immediately.

**Figure 1:**
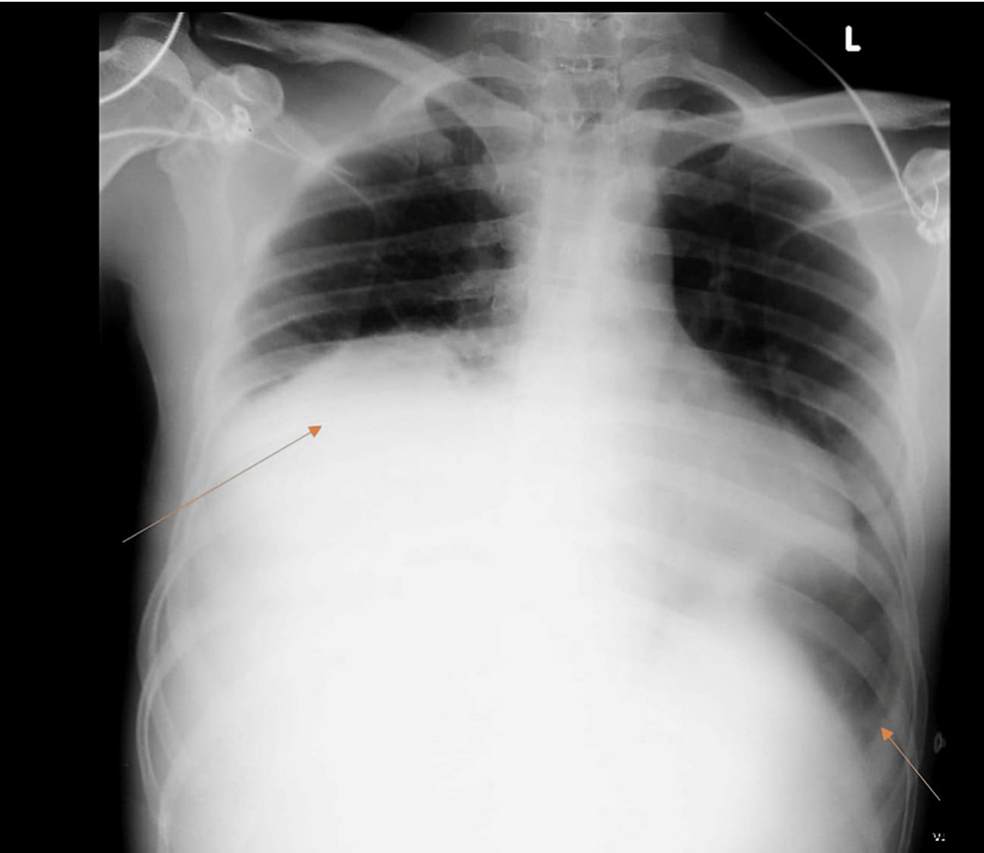
Chest X-ray in anteroposterior (AP) view shows moderate right pleural effusion and mild left pleural effusion (indicated by the arrows)

After the initial resuscitation, a chest X-ray was performed, which showed moderate right and mild left pleural effusion (Figure 1).

The blood evaluation of the patient is shown in Table 1.

**Table 1:** Investigations Boldface indicates abnormal values HIV and hepatitis A, B, C, D, and E serologies were negative Hb: hemoglobin; TLC: total leucocyte count; ESR: erythrocyte sedimentation rate; ALT: alanine transaminase; ALP: alkaline phosphatase; LDH: lactate dehydrogenase

Investigations	Patient values	Reference ranges
Hb	13.4 gm/dl	15.5 ± 2.5
TLC	33,700 per microlitre (mcL)	4,000–11,000
Platelets	68,000 per mcL	1,50,000–4,50,000
ESR	96 mm/hour	0–10 by Westergreen's method
Blood urea	70 mg/dl	7–20
Serum creatinine	0.9 mg/dl	0.6–1.2
Na+	106 mEq/l	135–145
Total bilirubin	11.61 mg/dl	0.2–1.0
ALT	278 IU/L	10–50
ALP	429 mg/dl	78–220
AST	583 IU/L	8–40
Albumin	1.8 g/dl	3.4–5
LDH	914 U/L	125–220

Urine evaluation of the patient showed urine protein 2+ and the presence of granular casts, which suggested renal parenchymal disease stage I.

**Figure 2:**
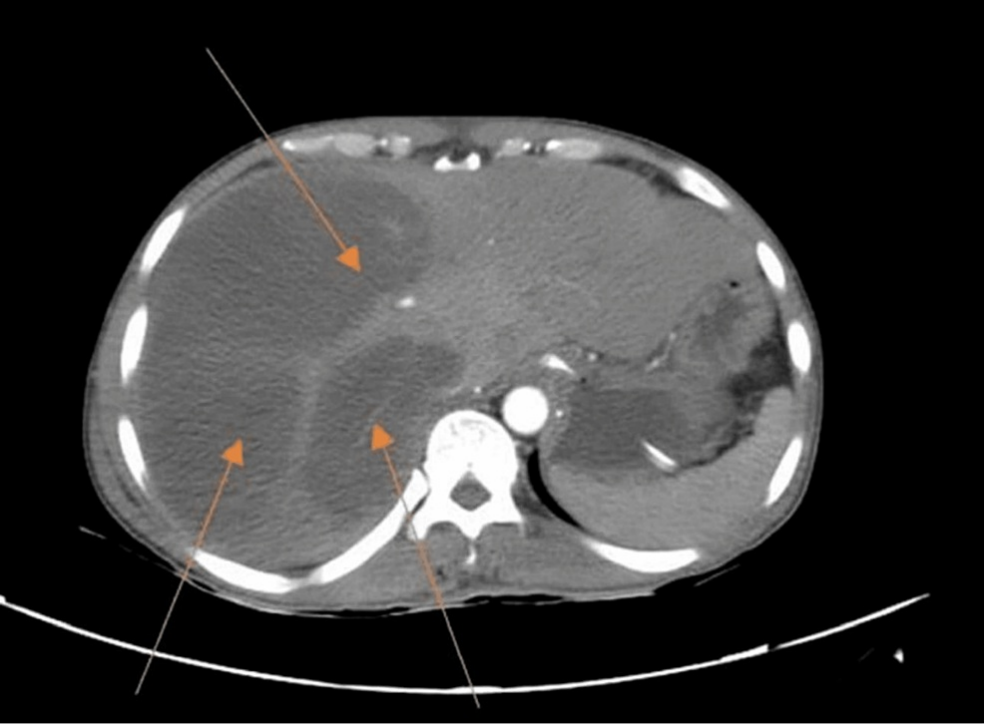
CT scan with IV contrast showing multiple liver abscesses (arrows) CT: computed tomography

**Figure 3:**
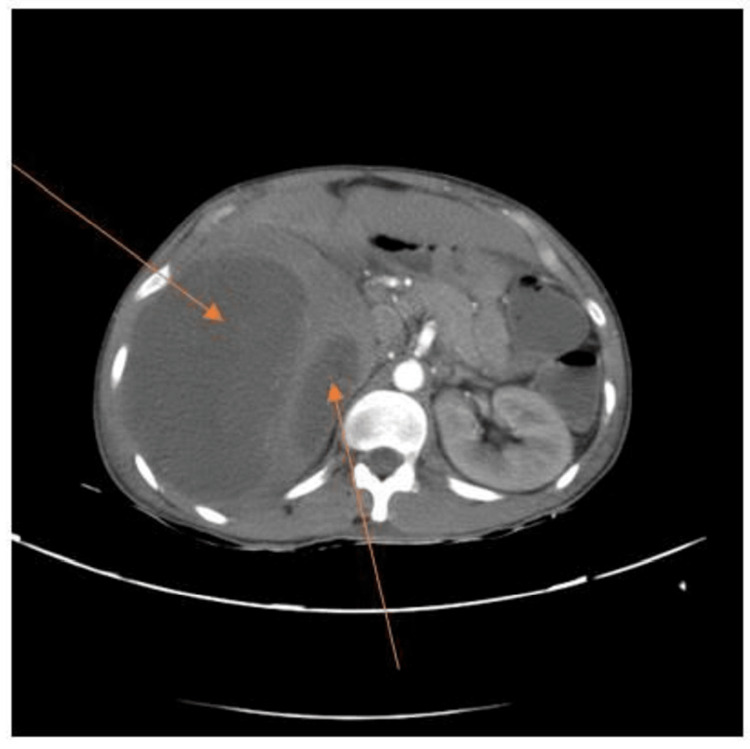
CT scan with IV contrast showing multiple liver abscesses extending from the right to the left lobe of the liver CT: computed tomography

**Figure 4:**
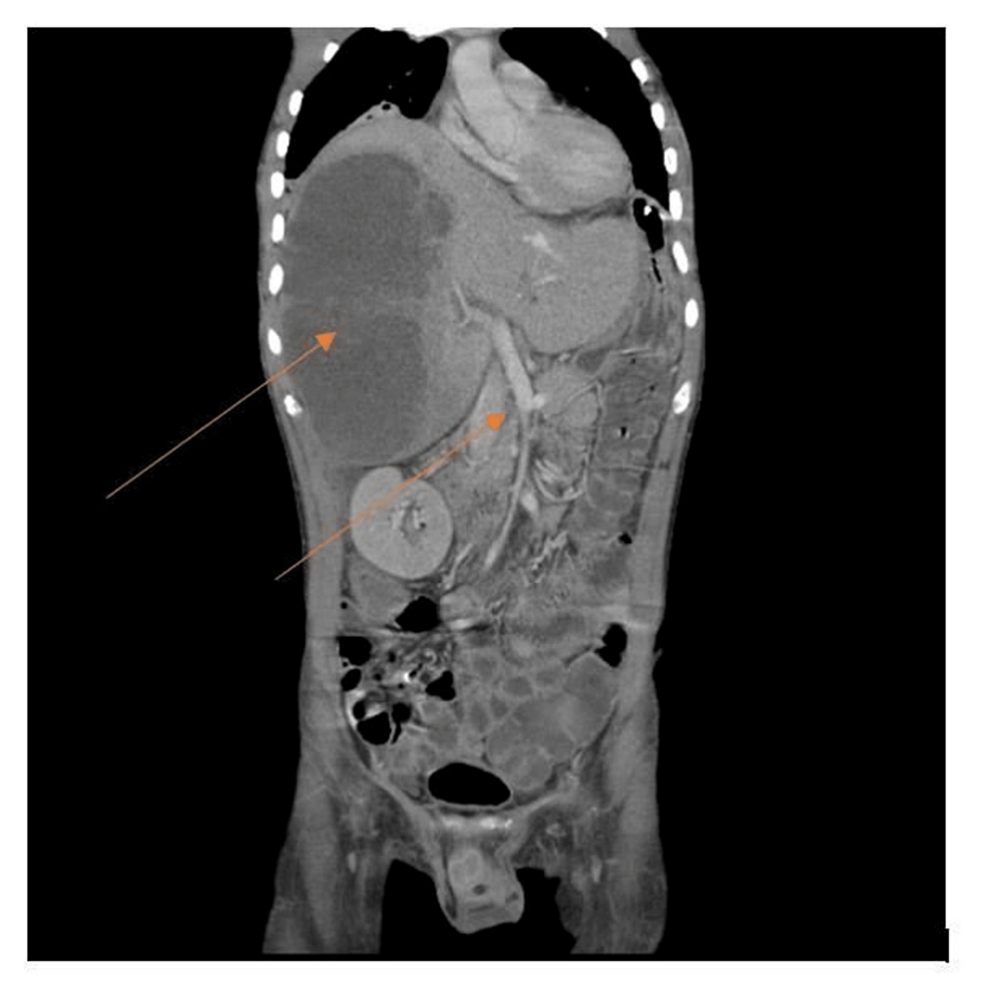
CT scan coronal view The image shows the enlarged liver compressing the gall bladder and right kidney, pushing it towards the psoas major muscle. The inferior vena cava has deviated from its normal course and appears to be compressing the portal vein and hepatic vein (indicated by the arrows) CT: computed tomography

Further evaluation was carried out by CT scan of the abdomen and pelvis, which indicated gross hepatomegaly with multiple well-defined multiloculated collections with the largest measuring 14.4 x 9.7 x 12.5 cm involving segments VII and VIII (Figures 2, 3) with another small lesion measuring 1.74 x 0.94 cm noted in the segment II. The enlarged liver seemed to be compressing the gall bladder and right kidney, pushing it towards the psoas major muscle (Figure 4). There was submucosal edema in the hepatic flexure of the colon (reactive edema to the inflammation caused by adjacent liver abscess/colitis). Serological tests were strongly positive for amoebic etiology.

During the hospital stay, the patient developed massive per rectal bleeding leading to hypotension (with a BP recording of 92/78 mmHg). A colonoscopy was performed, which showed crater-like cecal ulcers. Administration of fresh frozen plasma corrected the coagulopathy, thereby achieving optimal results.

**Figure 5:**
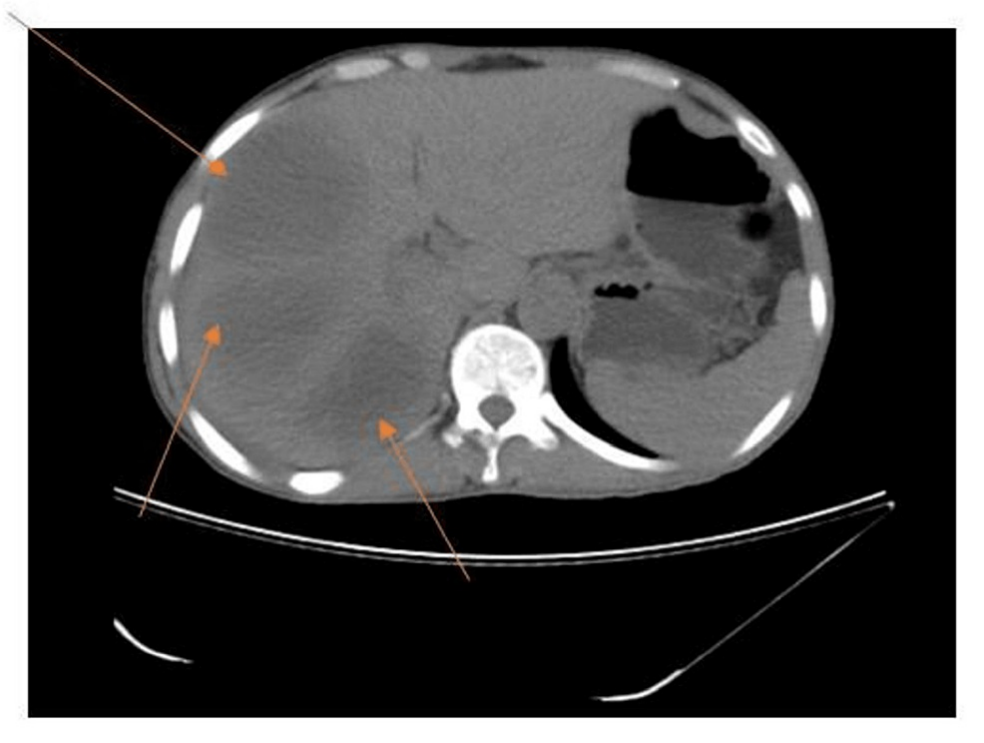
Repeat CT scan with IV contrast The image shows Hepatomegaly with large, thick-walled, peripherally enhancing collections showing few air pockets within the segments V, VI, VII, and VIII of the right lobe of the liver, only mildly reduced as compared with the previous collection (as shown by the arrows) CT: computed tomography

USG-guided percutaneous pigtail catheter insertion was carried out, revealing the characteristic "anchovy sauce" appearance of the drained pus. The specimen was sent for microscopic evaluation with modified trichrome stain and culture sensitivity, and the results showed the presence of amoebic trophozoites. Amoebic serology testing done by the ELISA method was positive, thereby confirming the radiological diagnosis. The patient was started on an intensive therapy of intravenous metronidazole 800 mg for 21 days. Pain in the abdomen decreased and jaundice subsided. The patient was discharged on maintenance anti-amoebic treatment after clinical improvement. However, the follow-up course within the next two weeks was further complicated by a persistent foul-smelling discharge at the catheter insertion site. A repeat CT scan showed hepatomegaly with large, thick-walled, peripherally enhancing collections in segments V, VI, VII, and VIII of the right lobe of the liver with only mild reduction compared to the previous collection (Figure 5). A hypoechoic tract with internal echoes was also noted, extending from the subcapsular to the overlying skin surface, measuring 2 cm.

The patient underwent excision of the granulation tissue and incision and drainage under general anesthesia with antimicrobial coverage for the above complaints. The tissue sent for histopathology was reported as s/o granulation tissue, and the pus from the abscess sent for culture and sensitivity showed no growth. After the surgery, the patient had persistent discharge from the site. A repeat ultrasound of the abdomen and pelvis review showed two liver abscesses in segments VI-VIII and a sinus tract from the subcapsular area to the skin. Positive amoebic serology was reported, and the patient was advised to undergo surgery, but he deferred it and got discharged against medical advice.

**Figure 6:**
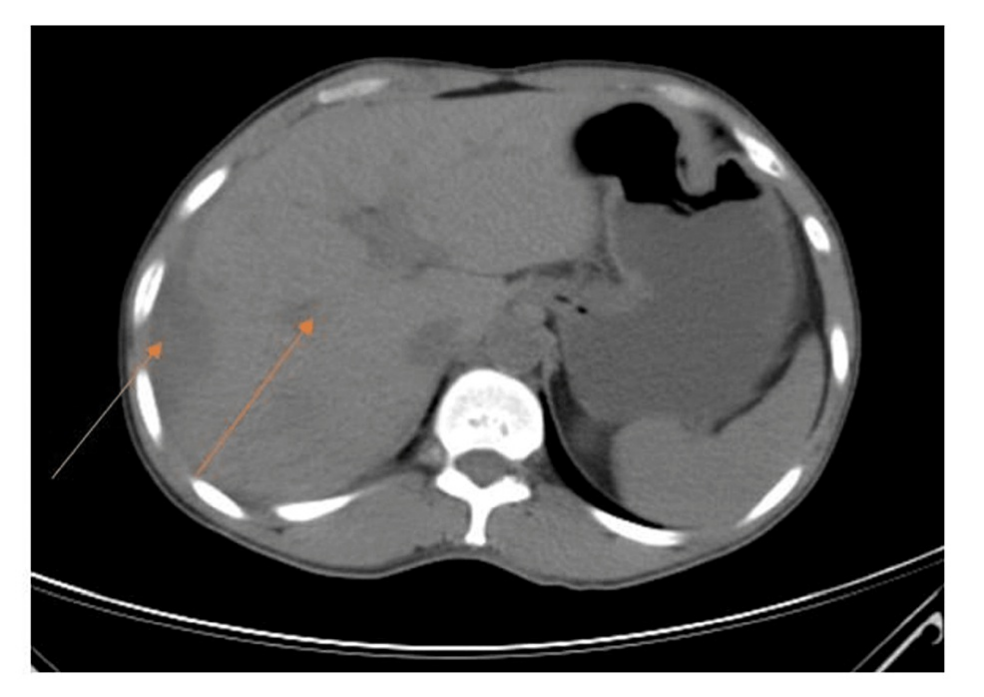
CECT abdomen and pelvis The image shows small organized abscesses in segment VI of the liver with adjacent mild fibrosis and scarring. No acute collections amenable to aspiration are seen in the present scan (shown by the arrows) CECT: contrast-enhanced computed tomography

The patient presented after two months with a complaint of black-colored loose stools (dysentery). The CECT abdomen and pelvis revealed small organized abscesses in segment VI of the liver with adjacent mild fibrosis and scarring (Figure 6), indicating chronic hepatic abscess with no acute collections amenable to aspiration. All medical evaluations, including routine investigations, were done and were found to be within normal limits [total leucocyte count, liver function tests, and C-reactive protein (CRP) were within normal limits]. After undergoing treatment with oral fluids and probiotics, the patient became asymptomatic and was subsequently discharged.

## Discussion

ALA can manifest in a bizarre manner, thereby creating a therapeutic dilemma. The variant of multiple ALA in our patient initially resulted in sepsis, only to persist with long-term sequelae despite adequate treatment. Treatment of complex multiloculated abscesses has not been studied extensively, and the condition usually does not resolve with medical management alone. This necessitates an evaluation of prognostic markers and conventional modalities available to re-access the management. The indicators of failure of medical management documented include the large size of the abscess (>10 cm), alkaline phosphatase levels >300 IU, and albumin levels <3 g/dl [7], which have been associated with poor to no response to therapy and require percutaneous drainage of the abscess leading to rapid resolution of the disease. However, while percutaneous catheter insertion in our patient led to clinical improvement of symptoms, he went on to develop a persistently draining sinus tract at the follow-up visit, revealing a residual disease with only a mild improvement in the abscess size.

This persuades us to explore other non-conventional treatment modalities documented in the medical literature that could have been employed to prevent the chronicity of the disease.

One of the options tested was drainage by Malecot catheter, used initially for percutaneous nephrostomy. The newer version is made of plastic (polyurethane) with an umbrella-shaped tip that balloons out when placed in the cavity, leading to better anchorage. Either blind insertion of the 10-12 Fr catheter in the eighth, ninth, or 10th intercostal space or the site guided by ultrasonography under local anesthesia using Seldinger's technique is performed. A study on 30 patients with an abscess size of more than 50 cc and existing risk factors like alcoholism and diabetes mellitus revealed a complete abscess resolution [8]. It was also associated with fewer complications, the most common being pain or local discomfort adequately controlled by analgesics. Blockage of the catheter was less frequent with 10 Fr catheters, even for thick pus. There was no associated ulcer, sinus, or fistula at the drain site after drain removal. Very few studies on the specific effects of Malecot catheter on liver abscesses have been published, and a survey conducted by Rehman et al. showed 100% success [9] with Malecot catheter with only minor complications. The study reported a 100% success rate with Malecot catheter drainage, which is more than the success rate of around 90-100% with pigtail catheters [9-10].

The insertion of multiple USG-guided catheters in the same sitting [11] is the second option mentioned in the medical literature. However, it is uncommon to insert several pigtail catheters into a single patient at once. According to Dulku et al. [12], three drainage treatments for three different abscesses were performed on one patient in one attempt, while two patients had two independent abscesses drained in the same sitting. Such instances are unusual and infrequent in the medical literature. The conventional route implicated surgical drainage for cases of multiple, large abscesses. This stereotype was busted by this article, taking into account that good patient selection and a meticulous approach led to avoidance of surgical intervention indicating multiple image-guided catheter drainage would suffice for complex abscesses. This could substantially decrease the monetary burden that surgery places on the patient as this disease is commonly prevalent among individuals in low socioeconomic strata. However, for a successful outcome, this study stressed careful follow-up and maintained antibiotic and metronidazole treatment even after discharge.

Laparoscopic drainage with a single incision is another minimally invasive surgical procedure. Laparoscopic drainage has been used in some cases with promising results, demonstrating a safer and viable alternative for patients who need surgical drainage after failing medical or percutaneous treatment, as well as those who have large or multiloculated abscesses with thick viscus pus or a cavity that is challenging to access by percutaneous means [12-13].

Lastly, there is an absolute need to treat patients with an intraluminal amebicide in invasive amebiasis. This was not carried out in our patient due to the unavailability of the procedure, leading to the formation of chronic hepatic abscess non-amenable to drainage, thereby increasing the risk of recurrence. The authors from the Amoebiasis Research Unit in Durban are credited for introducing and promoting the use of metronidazole to treat all forms of amoebiasis. Even though the drug has exceptional success rates in treating amoebic dysentery and amoebic liver abscess, it has not been effective in clearing cyst passers. Even with large doses of 800 mg three times daily, 15% of the cysts could not be removed. The authors found out that adding diloxanide furoate (Furamide) 500 mg to the routine regimen of metronidazole 400 mg taken thrice a day per oral for five days [14] proved efficacious in clearing cysts and luminal infections. In this case, the unavailability of luminal amebicides like diloxanide furoate put the patient at risk of residual amoebiasis.

## Conclusions

To sum up, an ALA can exhibit a variety of complications, necessitating the need for quick diagnosis and treatment. In cases where the patient fails to respond to conservative management and when the maximum diameter is greater than 10 cm, as in our case, a gradual step-up approach needs to be considered. If the patient declines to undergo surgery, non-conventional interventions such as Malecot drainage catheter, multiple pigtail catheter insertions in a single sitting, single incision laparoscopic surgery, and prescribing both luminal amebicide and extraluminal amebicides along with antibiotics for complete recovery should be considered so that full resolution of the disease is attained.

## Appendices

Reviewer questionnaire

(1) Summary of the research question:

Amoebic liver abscess is a prevalent disease in tropical regions, especially among alcoholics. However, few cases are irremediable through the commonly available medical or surgical modalities. Hence it seems imperative to explore and evolve newer not so commonly used techniques in tackling such complex cases to reduce the disease's long-term sequelae.

(2) Claims and how the research fits well in the existing literature?

The case report highlights the need for discovering and applying newer therapeutic methods in the treatment of the abscess if it fails to respond to the maximum medical dosage of the commonly used drug "Metronidazole" and the following best commonly used invasive method of pigtail tail drainage.

The studies referenced state better prognosis with techniques such as Malecot catheter drainage and the importance of diloxanide-metronidazole coadministration. Secondly, the surgical method of laparoscopic drainage rather than open drainage is better tolerated, associated with fewer complications, and decreases the overall surgical expenditure.

(3) Are the conclusions consistent with the evidence presented?

The conclusion provided by the study seems to be consistent with the evidence presented by various other authors who pondered the less traveled route to explore newer techniques to handle medically non-responsive cases.

(4) Obvious flaws or areas of weaknesses in the research:

The apparent flaw of the research is that none of the procedures mentioned has been employed to treat the given patient. The case ignites a spark to explore other therapeutic management that can halt the disease from developing long-term sequelae. Secondly, most cases respond well to the popularly used anti-amebicide "metronidazole" and pigtail catheter drainage, with only a few developing complicated sequelae. Therefore, this research article targets only those few patients; even the procedures carried out by other authors mentioned in the article have taken into account only a small sample population. Thus there is a need to practice and perfect these available techniques to prevent the residual disease from wreaking havoc on patients' health in the long term.
